# Compact Four-Port MIMO Antenna Using Dual-Polarized Patch and Defected Ground Structure for IoT Devices

**DOI:** 10.3390/s25144254

**Published:** 2025-07-08

**Authors:** Dat Tran-Huy, Cuong Do-Manh, Hung Pham-Duy, Nguyen Tran-Viet-Duc, Hung Tran, Dat Nguyen-Tien, Niamat Hussain

**Affiliations:** 1 Faculty of Electrical and Electronic Engineering, PHENIKAA University, Hanoi 12116, Vietnam; dat.tranhuy@phenikaa-uni.edu.vn (D.T.-H.); cuong.domanh@phenikaa-uni.edu.vn (C.D.-M.); hung.phamduy@phenikaa-uni.edu.vn (H.P.-D.); nguyen.tranvietduc@phenikaa-uni.edu.vn (N.T.-V.-D.); 2 Department of Convergence Engineering for Intelligent Drone, Sejong University, Seoul 13391, Republic of Korea; huyhung@sejong.ac.kr; 3 Department of Intelligent Mechatronics Engineering, Sejong University, Seoul 05006, Republic of Korea; niamathussain@sejong.ac.kr

**Keywords:** MIMO, dual-polarized patch, DGS, compact

## Abstract

This paper presents a compact four-port multiple-input multiple-output (MIMO) antenna for Internet-of-Things (IoT) devices. As electronic IoT devices become smaller, MIMO antennas should also be compact for ease of integration and multi-port operation for a high channel capacity. Instead of using a single-polarized radiator, which increases the antenna size when scaling to a multi-port MIMO array, a dual-polarized radiator is utilized. This helps to achieve multi-port operation with compact size features. To reduce the mutual coupling between the MIMO elements, an I-shaped defected ground structure is inserted into the ground plane. The measured results indicate that the final four-port MIMO antenna with overall dimensions of 0.92 λ× 0.73 λ× 0.03 λ at 5.5 GHz can achieve an operating bandwidth of about 2.2% with isolation better than 20 dB and a gain higher than 6.0 dBi. Additionally, the proposed method is also applicable to a large-scale MIMO array.

## 1. Introduction

Multiple-input multiple-output (MIMO) antennas have been used widely in Internet-of-Things (IoT) devices due to their ability to increase the channel capacity without the need for additional spectra [1]. The primary challenge in MIMO antenna design is suppressing mutual coupling, as strong coupling between the MIMO elements can degrade the system performance. Additionally, the other challenge is to maximize the number of MIMO ports while maintaining a compact antenna size. It is well-known that numerous MIMO antenna designs have been reported in the open literature, in which various antenna structures such as monopole, dipole, and microstrip patch configurations are employed. This paper specifically focuses on designing a MIMO antenna based on the microstrip patch structure, as it is suitable for compact, lightweight, and long-range communication systems.

The reported studies on MIMO patch antennas commonly consist of two or multiple patches arranged in H-plane or E-plane coupling configurations. To reduce mutual coupling, additional decoupling networks should be arranged properly. These could be a combination of decoupling and matching networks [2,3,4] or band-stop filters [5,6,7,8,9,10]. Alternatively, adding extra coupling paths to minimize the effect of the original path is also another effective solution [11,12,13,14,15]. Overall, the methodologies for designing these antennas are similar, in which the radiator produces single polarization, and it is excited by a single MIMO port. In this context, designing an n-port MIMO array requires n radiators, which consequently results in a significant increase in the antenna size.

To overcome the above-mentioned limitation, using dual-polarized radiators is a promising solution. In this scenario, the single radiator is fed by two exciting ports that have high inter-port isolation. As a result, the number of radiators required for an *n*-port MIMO array is significantly reduced to n/2, leading to a much more compact design compared to that in conventional approaches that use single-polarized radiators. This concept was investigated in [16,17,18,19], where dual-polarized square patches were utilized to design multi-port MIMO arrays. However, a critical drawback of these antennas is the large element spacing between the MIMO elements, leading to large antenna sizes.

This paper presents the design of a compact four-port MIMO antenna based on two dual-polarized patch radiators for a significant size reduction. To suppress mutual coupling between the MIMO elements, an I-shaped defected ground structure (DGS) is employed. Full-wave simulations conducted in High-Frequency Structure Simulator (HFSS) demonstrate the effectiveness of the proposed design, which is validated further through measurements. Moreover, the investigation suggests that this approach can be extended to large-scale MIMO arrays, highlighting its scalability. In comparison with other related MIMO designs using dual-polarized radiators [16,17,18,19], the proposed designs has advantages in both its compactness and isolation.

## 2. The Dual-Polarized Patch Antenna

**Figure 1:**
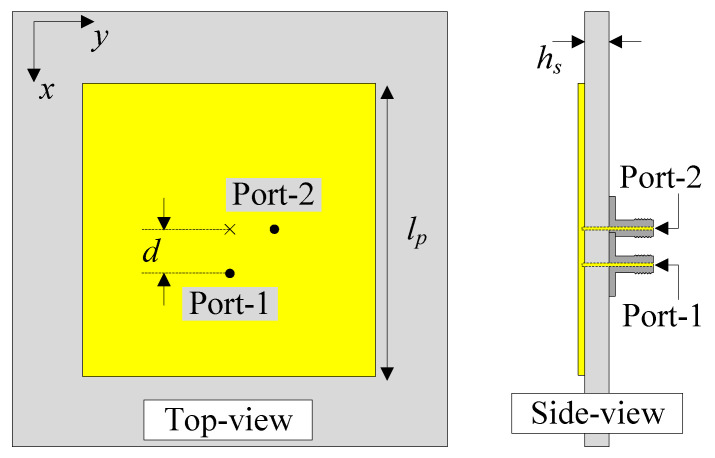
The geometry of the dual-polarized patch antenna.

Figure 1 illustrates the geometry of the proposed dual-polarized patch antenna, including both top and side views. The Taconic TLY-5 material with a dielectric constant of 2.2 is utilized as the antenna’s substrate. Dual-polarized operation is achieved through orthogonal feeding, with Port-1 oriented along the *x*-axis and Port-2 positioned along the *y*-axis. When the patch is excited at Port-1, a null locus will be produced in the y-direction, which will result in high inter-port isolation. Equally, as the impedance of the patch varies continuously from 0 Ω at the center to infinity at the edges, the feeding position is tuned within this range. Perfect matching will be achieved at the position where the impedance of the patch is 50 Ω. The optimal dimensions of the proposed antenna are as follows: $l_{p}=17$ mm, d=2.8 mm, and $h_{s}=1.52$ mm.

**Figure 2:**
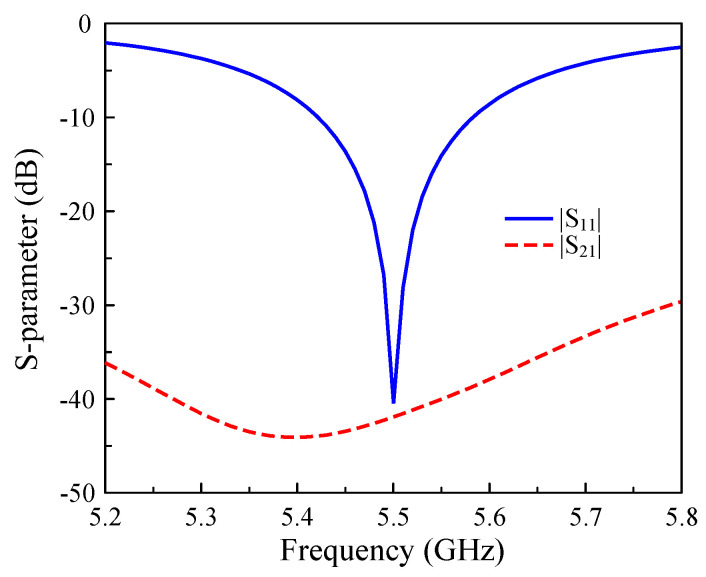
Simulated S-parameters of the dual-polarized patch antenna.

Figure 2 presents the simulated S-parameters of the dual-polarized antenna, demonstrating its excellent performance. The antenna offers optimal impedance matching at 5.5 GHz, with a reflection coefficient below −10 dB across the operational bandwidth from 5.42 GHz to 5.58 GHz. Notably, an exceptional inter-port isolation of approximately 40 dB is achieved at the design frequency, indicating minimal coupling between the orthogonally positioned Port-1 and Port-2.

## 3. The Four-Port MIMO Antenna Without a Decoupling Network

**Figure 3:**
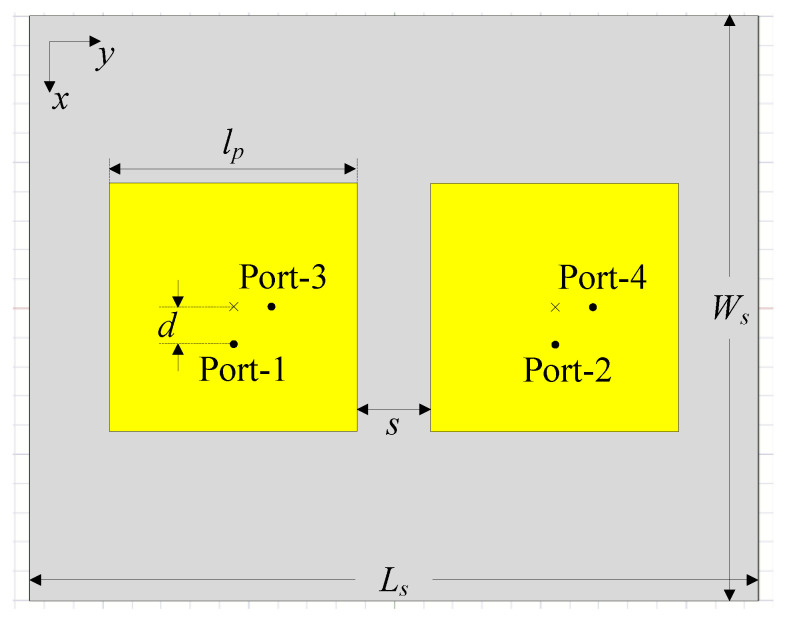
The geometry of the four-port MIMO antenna without a decoupling structure.

In this section, a compact four-port MIMO array configuration is developed based on the dual-polarized antenna design presented in the previous section. At this stage, the operation of the four-port coupled MIMO antenna is thoroughly investigated. As depicted in Figure 3, the array configuration consists of two dual-polarized patch elements with a center-to-center spacing of 0.4λ, where λ represents the free-space wavelength at 5.5 GHz. The design parameters are as follows: $l_{p}=17$ mm, d=2.8 mm, s=5 mm, $L_{s}=50$ mm, and $W_{s}=40$ mm.

**Figure 4:**
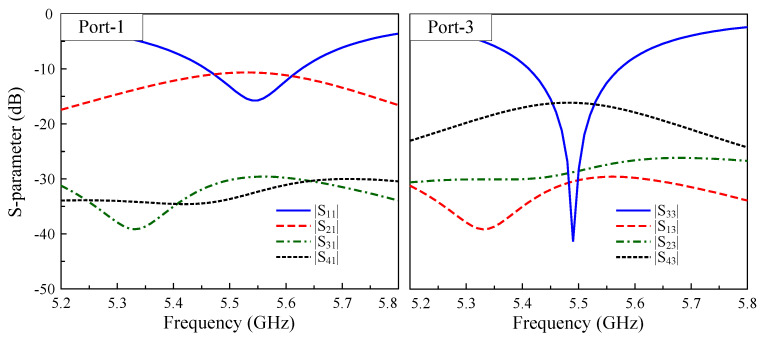
Simulated S-parameters of the 4-port MIMO antenna without a decoupling structure.

Figure 4 shows the simulated S-parameters of the four-port MIMO array in its baseline configuration without decoupling networks. The results reveal that while the array maintains good impedance matching, it still suffers from remarkably high mutual coupling between elements. The lowest isolation between Port-1 and -2 (H-plane coupling) is about 10 dB, while this figure for Port-3 and -4 (E-plane coupling) is about 16 dB. That is to say, a coupling effect occurs in both the E-plane and H-plane orientations. Therefore, a decoupling network capable of the suppressing mutual coupling in both planes is essential.

## 4. The Four-Port MIMO Antenna with a Decoupling Network

While numerous decoupling structures have been proposed in the literature, most of them demonstrate effectiveness in reducing coupling either in the H-plane or the E-plane for patch-based MIMO systems. The proposed work addresses this limitation through the implementation of an I-shaped defected ground structure (DGS). As demonstrated in subsequent sections, this approach simultaneously suppresses the mutual coupling in both the E- and H-planes, offering a comprehensive solution for dual-polarized MIMO arrays.

### 4.1. H-Plane Decoupling

**Figure 5:**
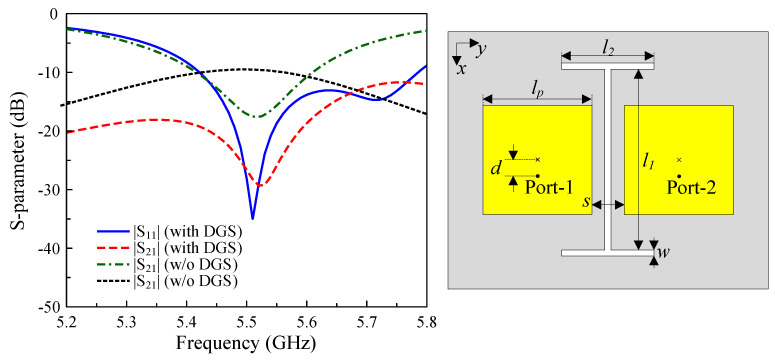
The geometry and S-parameters of the H-plane coupled MIMO antenna with the I-shaped DGS.

Figure 5 exhibits the geometry of the utilized I-shaped DGS deployed between two square patches in the H-plane coupled configuration. The key design parameters are optimized as follows: $l_{p}=16.8$ mm, d=3.4 mm, s=5 mm, w=1 mm, $l_{1}=27.6$ mm, and $l_{2}=15$ mm. The comparative results on the S-parameter in Figure 5 demonstrate that while both the configurations with and without the DGS maintain good impedance matching at 5.5 GHz, the DGS-based design produces significantly improved isolation. Specifically, the isolation reaches 30 dB, representing an enhancement of 13 dB at the desired frequency. It is also worth noting that the DGS breaks the current distribution symmetry, which leads to unwanted radiation in the orthogonal polarization components. Accordingly, the antenna with the DGS suffers from a high level of cross-polarization and high back radiation.

**Figure 6:**
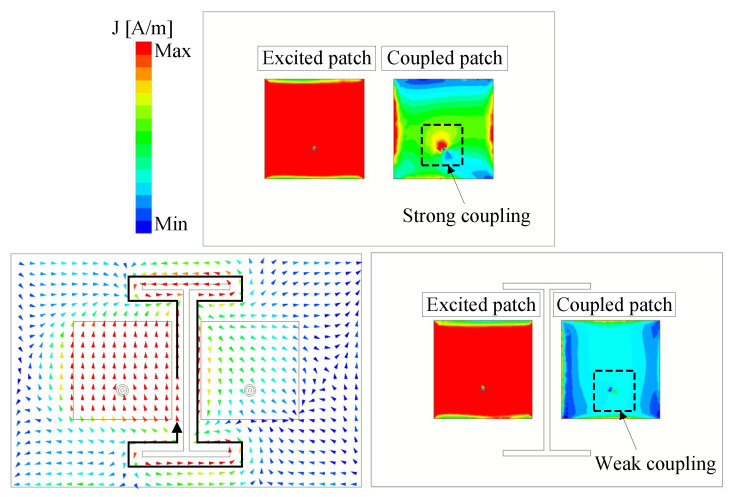
Simulated current distribution at 5.5 GHz.

The implemented I-shaped DGS effectively mitigates mutual coupling by controlling the surface currents between antenna elements. Figure 6 clearly demonstrates this mechanism through the simulated current distributions at 5.5 GHz for both configurations, with and without the DGS. For the design without the DGS, strong coupling currents flow directly between the excited and passive patches, causing a poor isolation performance. In contrast, the DGS-modified design shows these currents being driven along the I-shaped defect structure. In this scenario, the DGS functions as a band-stop filter, simultaneously diverting the coupling currents away from the passive element and dissipating their energy through the engineered defect pattern. This dual-action mechanism results in significantly improved inter-element isolation.

**Figure 7:**
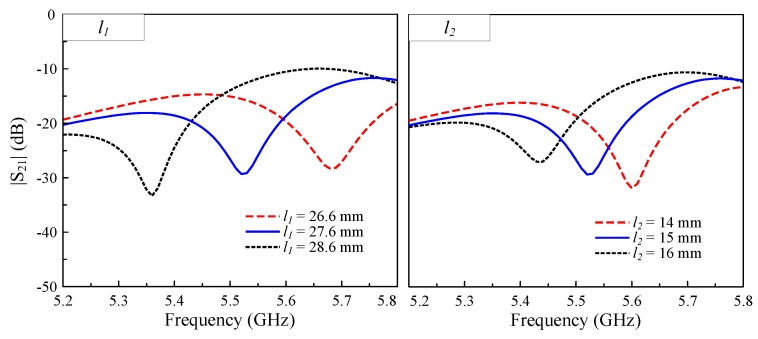
Simulated $|S_{21}|$ values for different lengths of the I-shaped DGS.

The isolation levels, as evidenced by the dip in the transmission coefficient, can be precisely controlled through dimensional optimization of the I-shaped DGS. Figure 7 presents a parametric study of $|S_{21}|$ versus the frequency for varying lengths $l_{1}$ and $l_{2}$, in which the corresponding $|S_{11}|$ results are omitted, as these parameters show a negligible impact on the impedance matching. Three key observations can be made from the simulated results. Firstly, both $l_{1}$ and $l_{2}$ significantly affect the isolation bandwidth and depth. The second point is that increasing either dimension causes the isolation notch to shift toward lower frequencies. Lastly, the tuning range demonstrates consistent and predictable behavior, which is suitable for design optimization. This tunability allows for targeted suppression of the mutual coupling at the desired operational frequencies while maintaining a stable impedance matching performance.

### 4.2. E-Plane Decoupling

**Figure 8:**
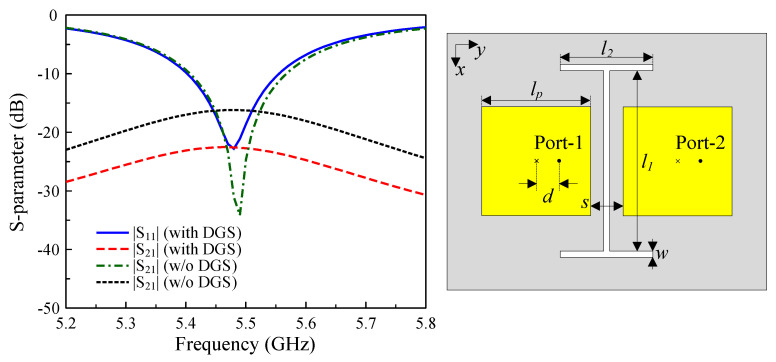
The geometry and S-parameter of the E-plane coupled MIMO antenna with an I-shaped DGS.

Figure 8 demonstrates the role of the I-shaped DGS in reducing the mutual coupling for E-plane coupled MIMO patch antennas. For the case without any decoupling structure, the E-plane coupled patches exhibit relatively poor isolation of approximately 16 dB. With the deployment of the I-shaped DGS, the isolation improves significantly to 23 dB, representing a 7 dB enhancement. It is noteworthy that both configurations maintain identical element spacing, although the improvement in the E-plane coupled configuration is less significant than that achieved in the H-plane.

### 4.3. Final Realization of the Four-Port MIMO Antenna

**Figure 9:**
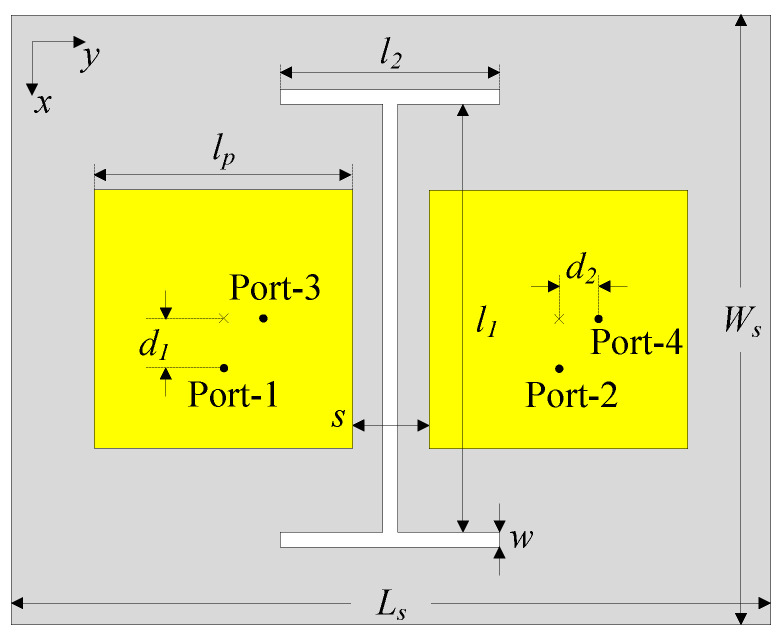
The geometry of the proposed 4-port MIMO antenna with the I-shaped DGS.

**Figure 10:**
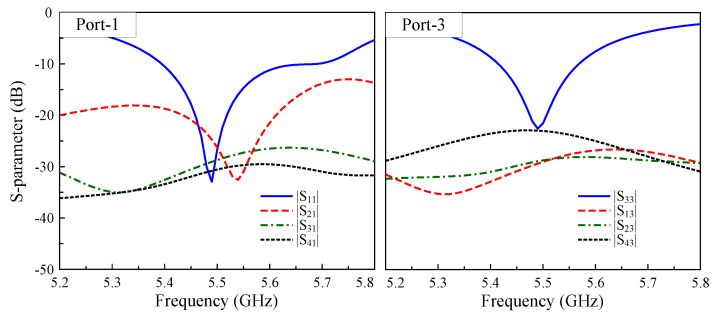
Simulated S-parameters of the proposed 4-port MIMO antenna.

The final realization of the proposed four-port MIMO array is depicted in Figure 9. The antenna’s optimal dimensions are as follows: $l_{p}=17$ mm, $d_{1}=3.4$ mm, $d_{2}=2.8$ mm, s=5 mm, w=1 mm, $l_{1}=28$ mm, $l_{2}=14.4$ mm, $L_{s}=50$ mm, and $W_{s}=40$ mm. It is worth noting that the feeding positions of Port-1, -2 and Port-3, -4 are slightly offset due to the different coupling scenarios. The S-parameter results when the antenna operates with Port-1 and Port-3 are illustrated in Figure 10. The simulated results demonstrate an excellent performance in terms of the impedance matching and port isolation, in which the shared impedance bandwidth of both ports is from 5.42 to 5.57 GHz. Over this band, the isolation among all ports is always greater than 23 dB, which is much better than that for the antenna without the DGS discussed in the previous section.

**Figure 11:**
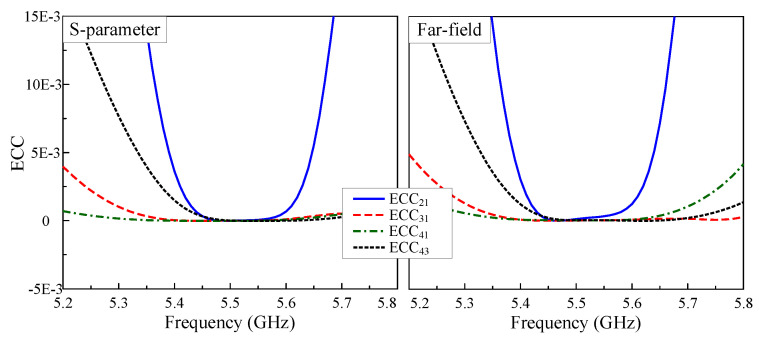
The calculated ECC of the proposed 4-port MIMO antenna.

To verify the adaptability of the proposed four-port antenna to MIMO systems, its diversity performance should be quantitatively assessed, in which a vital metric is the Envelope Correlation Coefficient (ECC). This parameter can be calculated based on the S-parameter or the far-field radiation pattern, as described in Equations (1) and (2). Figure 11 shows the ECC results achieved through both methods of calculation, demonstrating remarkable consistency between both approaches. Notably, the array offers excellent ECC values below 0.002 at around 5.5 GHz, significantly outperforming the benchmark 0.5 threshold for practical MIMO systems. These outstanding results confirm the array’s superior diversity performance.

(1) $$ECC_{ij}=\frac{{\left|S_{ii}^{*}S_{ij}+S_{ji}^{*}S_{jj}\right|}^{2}}{\left(1-{\left|S_{ii}\right|}^{2}-{\left|S_{ji}\right|}^{2}\right)\left(1-{\left|S_{jj}\right|}^{2}-{\left|S_{ij}\right|}^{2}\right)}$$

(2) $$ECC_{ij}=\frac{{\left|{\;\int \int \;}_{4\pi }\left({\vec{B}}_{i}\left(\theta ,\phi \right)\right)\times \left({\vec{B}}_{j}\left(\theta ,\phi \right)\right)d\Omega \right|}^{2}}{{\;\int \int \;}_{4\pi }{\left|\left({\vec{B}}_{i}\left(\theta ,\phi \right)\right)\right|}^{2}d\Omega {\;\int \int \;}_{4\pi }{\left|\left({\vec{B}}_{j}\left(\theta ,\phi \right)\right)\right|}^{2}d\Omega }$$

## 5. A Multi-Port MIMO Array

**Figure 12:**
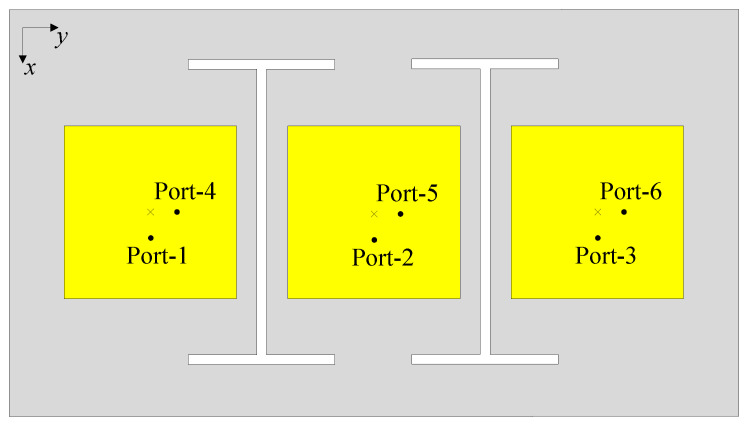
The geometry of the 6-port MIMO antenna with I-shaped DGSs.

**Figure 13:**
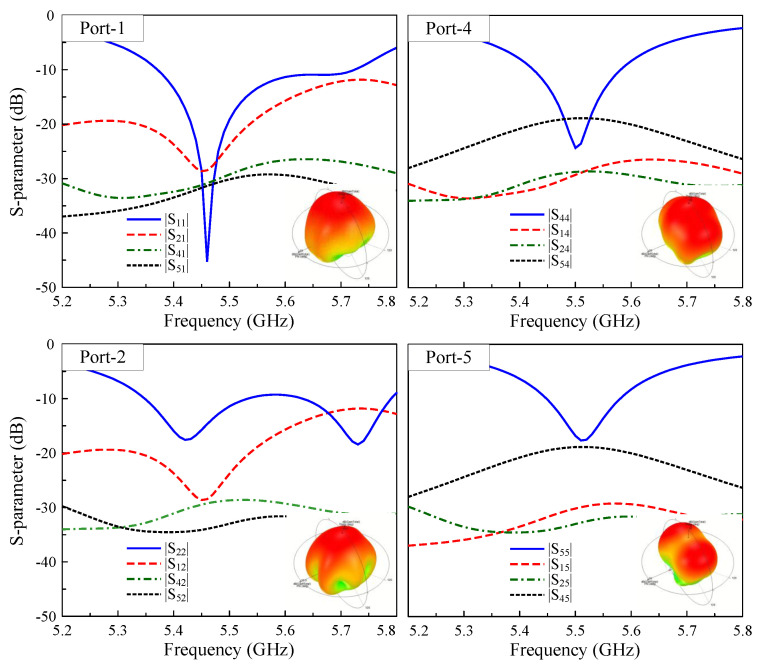
Simulated S-parameters and 3-D radiation patterns of the 6-port MIMO antenna with different port operation.

To validate the scalability of the proposed methodology, the design is extended to a six-port MIMO array comprising three dual-polarized radiators with two integrated I-shaped DGSs, as depicted in Figure 12. The simulated S-parameters for Port-1, -2, -4, and -5 and the corresponding 3-D radiation patterns at 5.5 GHz are illustrated in Figure 13. It can be concluded from the results that the matching performance with different port excitations is uniform, with reflection coefficients consistently below −10 dB around 5.5 GHz. In addition, the isolation levels among all ports are always high, exceeding 18 dB. To conclude, the consistent performance across the four- and six-port implementations confirms that the proposed design approach is applicable to multi-port MIMO arrays, where the MIMO elements are arranged into one-dimensional structures.

## 6. Measurements

**Figure 14:**
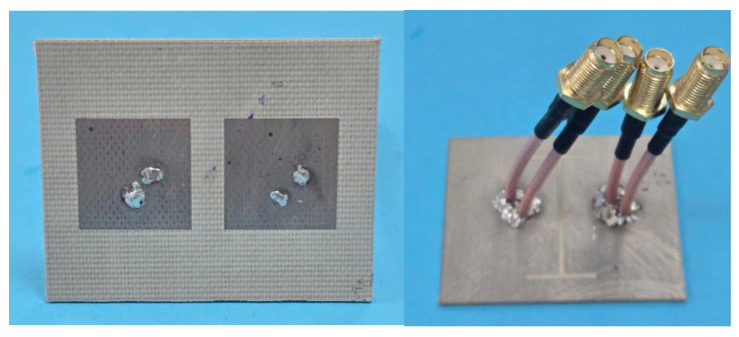
Photographs of the fabricated antenna prototype.

Figure 14 illustrates the prototype of the proposed antenna, fabricated to validate the design concept experimentally. The prototype was characterized through S-parameter measurements using a vector network analyzer (VNA) and far-field radiation measurements in an anechoic chamber. While the measured results show excellent agreement with the simulations overall, minor discrepancies are observed, primarily attributable to fabrication tolerances in the PCB manufacturing process and inevitable imperfections in the measurement environment. These variations fall within the acceptable margins for practical implementation and do not affect the antenna’s core performance characteristics.

**Figure 15:**
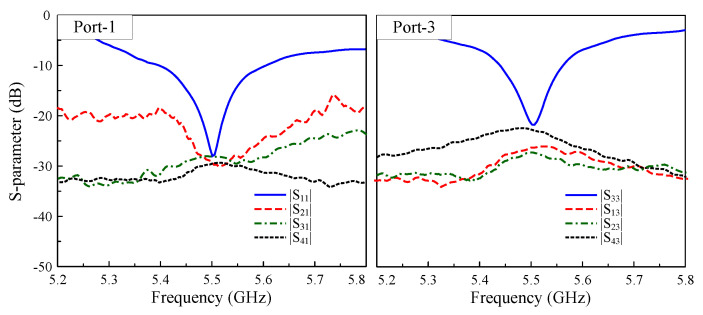
Measured S-parameters of the proposed 4-port MIMO antenna.

**Figure 16:**
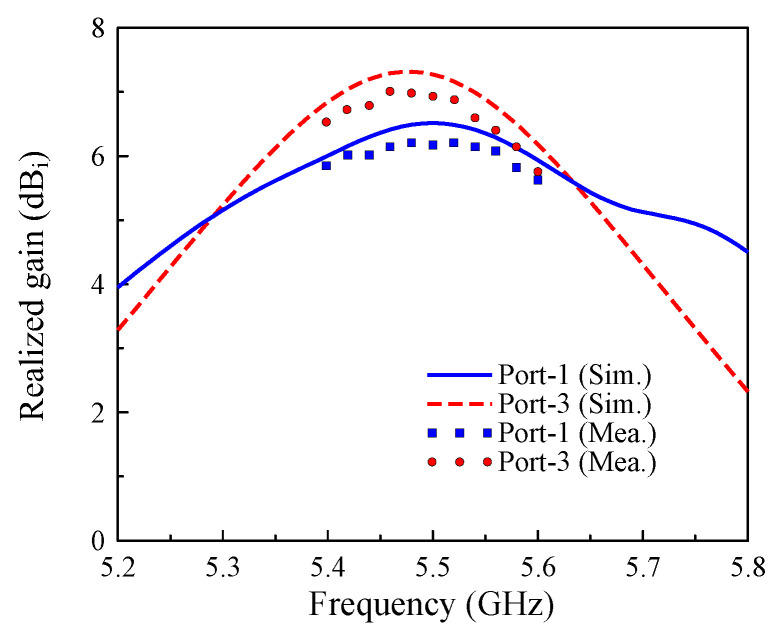
Simulated and measured gain of the proposed 4-port MIMO antenna.

**Figure 17:**
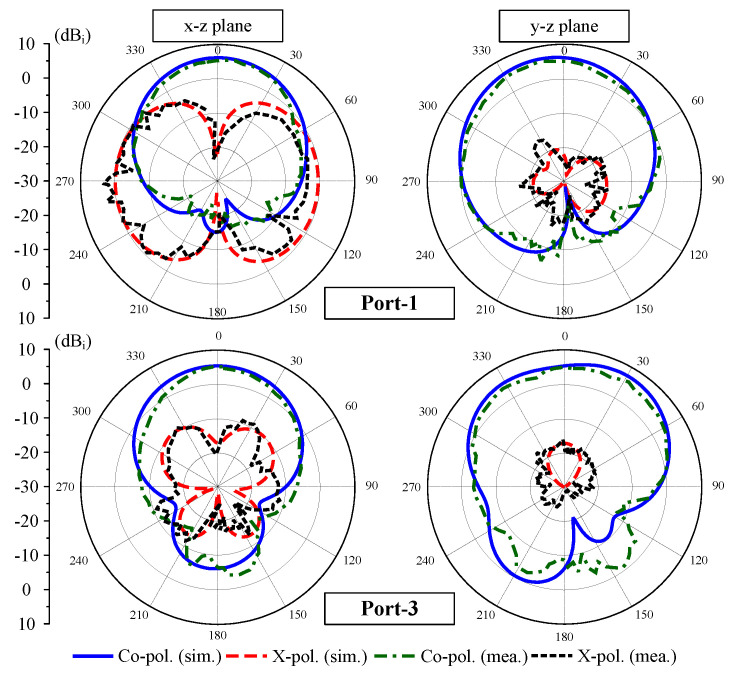
Simulated and measured gain radiation patterns at 5.5 GHz for the proposed 4-port MIMO antenna.

The measured performance of the prototype is presented in Figure 15, Figure 16 and Figure 17. Figure 15 demonstrates excellent agreement between Port-1 and Port-3, showing an overlapped impedance bandwidth of 2.2% from 5.44 GHz to 5.56 GHz with consistent isolation exceeding 20 dB across the operational band. It is revealed from Figure 16 that the four-port MIMO antenna produces a stable realized gain of above 6.0 dBi throughout the bandwidth, reaching a peak gain of 7.0 dBi under Port-1 excitation. The radiation pattern measured at 5.5 GHz, as depicted in Figure 17, further validates the design, exhibiting consistent results between the simulation and measurements, particularly in the main beam direction.

A comparison between the proposed MIMO antenna and other recently reported MIMO antenna designs is summarized and depicted in Table 1. A quick glance at this table highlights two key benefits of the proposed approach, which are the significant size reduction achieved through the optimized dual-polarized radiator configuration and the scalable multi-port operation without compromising the isolation performance. Compared to the single-polarized designs in [12,13], the use of dual-polarized radiators doubles the number of operating ports while maintaining comparable overall dimensions. When evaluated against other dual-polarized arrays [16,17,18,19], the proposed designs offers a superior performance in both its compactness and isolation.

## 7. Conclusions

A MIMO antenna with four-port operation and compact size characteristics has been presented in this paper. The proposed approach utilizes dual-polarized radiators and an I-shaped DGS to achieve both a compact size and high isolation. This study begins with a comprehensive analysis of a four-port MIMO configuration, followed by extension to a six-port array to validate the scalability of the proposed design methodology for large-scale MIMO systems. The design concept is validated by measurements, which show an operating bandwidth of 2.2% with isolation better than 20 dB and a gain greater than 6.0 dBi. The proposed antenna possessing a multi-port operation capability, high isolation, and a compact-size structure makes it suitable for modern IoT devices.

## Figures and Tables

**Table 1:** Performance comparison among MIMO patch antennas.

Ref.	Overall Dimensions (λ)	Decoupling Scheme	MIMO Element	No. of Ports	No. of Layers	Spacing (λ)	Isolation (dB)
[12]	1.26 × 0.84 × 0.02	Extra coupling path	Single-pol.	2	1	0.4	≥15
[13]	0.88 × 0.58 × 0.03	Extra coupling path	Single-pol.	2	1	0.3	≥20
[16]	1.07 × 0.55 × 0.02	Band-stop filter	Dual-pol.	4	2	0.42	≥10
[17]	1.03 × 0.52 × 0.10	Extra coupling path	Dual-pol.	4	2	0.51	≥15
[18]	1.38 × 0.69 × 0.10	Cavity for field blocking	Dual-pol.	4	2	0.53	≥20
[19]	1.47 × 1.74 × 0.05	Matching network	Dual-pol.	4	1	0.5	≥15
Prop.	0.92 × 0.73 × 0.03	Band-stop filter	Dual-pol.	4	1	0.4	≥20

## Data Availability

The data are contained within the article.
